# mSphere of Influence: the Complexity of Interferon Gamma-Mediated Pathogen Control

**DOI:** 10.1128/mSphere.01295-20

**Published:** 2021-01-06

**Authors:** Bryan D. Bryson

**Affiliations:** a Department of Biological Engineering, Massachusetts Institute of Technology, Cambridge, Massachusetts, USA; b Ragon Institute of MGH, MIT, and Harvard, Cambridge, Massachusetts, USA

**Keywords:** *Legionella pneumophila*, interferon gamma, macrophage

## Abstract

Bryan D. Bryson works in the field of biological engineering with a specific interest in host-mycobacterium interactions. In this mSphere of Influence article, he reflects on how “IRG1 and inducible nitric oxide synthase act redundantly with other interferon-gamma-induced factors to restrict intracellular replication of Legionella pneumophila” by Price and colleagues (J. V. Price, D. Russo, D. X. Ji, R. A. Chavez, et al., mBio 10:e02629-19, 2019, https://doi.org/10.1128/mBio.02629-19) made an impact on him by reinforcing the complexity of intracellular pathogen control.

## Abstract (precis)

Bryan D. Bryson works in the field of biological engineering with a specific interest in host-mycobacterium interactions.

## COMMENTARY

Control of phagosomal pathogens by innate immune cells both fascinates and confuses me at the same time. My fascination with immune control of pathogens is born out of the diversity of pathogens that immune cells must confront and ultimately eliminate. On the other hand, I find pathogen control confusing at times given the sheer number of proposed antimicrobial mechanisms and our limited understanding of how these antimicrobial mechanisms synergize or antagonize one another (1). The phagosome is an undisputed cornerstone of the interaction between host and pathogen; however, how this dynamically formed organelle coordinates the appropriate biochemistry required for pathogen restriction or killing remains incompletely understood. A common mechanism often invoked to explain how control of intracellular pathogens is achieved involves the induction of inducible nitric oxide synthase (iNOS); however, as Price et al. beautifully demonstrate in reference 2, this model falls short in explaining the observed relationship between interferon gamma (IFN-γ) treatment, host genotype, and control of Legionella pneumophila (3–5). Using a series of genetic knockouts, Price et al. demonstrate that six genes contribute to the totality of IFN-γ-mediated control and thus expand the mechanistic basis for control of intracellular bacteria.

Using a series of mutant macrophages lacking genes such as iNOS, ATG5, and guanylate-binding proteins (GBPs) infected with a luminescent strain of *Legionella*, Price et al. demonstrated that loss of individual genes did not abrogate IFN-γ-mediated L. pneumophila control. The authors next made use of small-molecule perturbation studies and gene expression profiling to test hypotheses related to IFN-γ-mediated metabolic remodeling and the unfolded protein response. These experiments did not reveal a unifying model to explain how IFN-γ enhances L. pneumophila control. Price et al. returned to gene expression data in conjunction with their L. pneumophila growth experiments in the presence of distinct small molecules to identify transcriptional programs associated with permissive or restrictive macrophage states. Using this approach, the authors identified an association between *Acod1* (which encodes the protein Irg1 that contributes to itaconate production) expression and *Nos2* expression and macrophage antimicrobial states (6, 7). The authors next generated macrophages lacking *Acod1* and again determined that loss of this gene in isolation did not abrogate IFN-γ-mediated control. The authors next turned to higher-order mutations and ultimately demonstrated that deletion of six genes was required to fully disrupt interferon IFN-γ-mediated control.

For me, this paper confirmed a model that I had always believed to be true but did not have the data to support. I remember beginning my postdoctoral training trying to learn as much as possible about the antimicrobial functions of phagocytes; however, I remember finding myself making a list of the many antimicrobial mechanisms implicated in bacterial control ranging from oxidative radicals to acidification to nutritional immunity and asking how these antimicrobial mechanisms work in concert (8–10). It always made intuitive sense that a cell would have multiple redundant antimicrobial mechanisms in order to ensure effective pathogen clearance. Many of the studies I had read had focused exclusively on a specific antimicrobial pathway without analyzing the induction of the litany of additional antimicrobial mechanisms. For some of these studies, similar to the observations of Price and colleagues, loss of specific genes in these studies demonstrated that loss of an individual gene partially restored bacterial growth and survival. What is accounting for the residual pathogen control in an activated phagocyte lacking a key antimicrobial gene? To me, this paper underscores the importance of characterizing a broad and diverse array of antimicrobial programs induced or disrupted in the setting of enhanced or worsened pathogen control. As a biological engineer, these studies encouraged me and my group to deepen our characterization and identification of the diverse array of antimicrobial programs that contribute to bacterial control before ascribing enhanced control to a specific mechanism. Additionally, the observed partial loss of bacterial control via genetic mutants has also encouraged us to deepen our characterization of the phagosome—does the partial abrogation of bacterial control reflect subpopulation dynamics or do all phagosomes acquire similar properties during activation?

Looking ahead, I am eager to see how this model generalizes to additional pathogens and phagocytes. Are the six genes identified by Price and colleagues similarly required for control of other pathogens whose control is enhanced by IFN-γ? Notably, iNOS is robustly detected in murine macrophages following activation with IFN-γ; however, detection of iNOS in human macrophages has been more challenging and controversial. Do human macrophages have similar redundancies? Are these six genes required for control in other restrictive macrophage settings induced by other cytokines or small molecules? The number of questions raised by this simple but elegant study will undoubtably invigorate our collective thinking about the complexity of antimicrobial control and the diverse paths that immune cells traverse to protect us from some of the world’s most deadly pathogens.
